# Supplementary material for: Systematic characterization of pan‐cancer mutation clusters
Source: Mol Syst Biol. 2018 Mar 25;14(3):e7974. doi: 10.15252/msb.20177974 (PMC5866917; doi:10.15252/msb.20177974)
Supplement: Supplementary file 1 — Expanded View Figures PDF [file MSB-14-e7974-s001.pdf]

Expanded View Figures

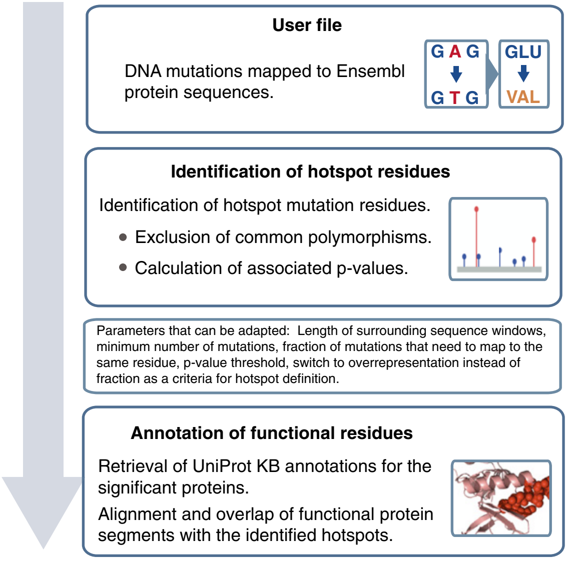

Figure EV1. *DominoEffect* R package.

We used the in-house Perl script to map genomic mutations onto protein sequences, and we are happy to share it upon request. The obtained mutation dataset is then be analyzed with the *DominoEffect* R package. In the first step, the package identifies hotspot mutation residues and in the second step it obtains UniProt-SwissProt KB annotations for the respective proteins and provides information on the protein functional segment affected by the hotspot mutation. In the latter step, it also assures that the Ensembl and UniProt sequence alignments are in agreement. When identifying hotspot residues, the package allows the user to flexibly change different parameters. Hotspot identification and residue annotation can be run separately or jointly by calling the *DominoEffect* function. The package provides a test mutation dataset and comes with a vignette that explains the options in detail.

© 2018 The Authors
