# Supplementary material for: Systematic characterization of pan‐cancer mutation clusters
Source: Mol Syst Biol. 2018 Mar 25;14(3):e7974. doi: 10.15252/msb.20177974 (PMC5866917; doi:10.15252/msb.20177974)
Supplement: Supplementary file 2 — Code EV1 [file MSB-14-e7974-s002.zip › DominoEffect/vignettes/Vignette.html]

Vignette for DominoEffect package


# Vignette for DominoEffect package

#### *Marija Buljan and Peter Blattmann*

#### *12 February 2018*

The package can be directly installed from the Bioconductor website.

```
## try http:// if https:// URLs are not supported
source('https://bioconductor.org/biocLite.R')
biocLite('DominoEffect')
```

## Running the package

Load the package before starting the analysis.

```
library(DominoEffect)
```

The package provides example datasets to run the functions.

```
data("TestData", package = "DominoEffect")
data("SnpData", package = "DominoEffect")
data("DominoData", package = "DominoEffect")
```

## Optional

The default settings are to use the package-provided data that will be loaded in the objects mutation\_dataset, gene\_data and snp\_data, respectively. Required column names are described below and the user should adher to these when providing their own mutation dataset. Gene information (DominoData) and SNP data (SnpData) correspond to the Ensembl version 73 (GRCh37). Optionally, the user can use data for different Ensembl releases and provide larger SNP datasets.

```
mutation_dataset = read.table ("user_file_with_mutations.txt", header = T)
gene_data = read.table ("user_ensembl_gene_list.txt", header = T)
snp_data = read.table ("user_population_SNPs_with_frequency.txt", header = T)
```

## Description

The package identifies individual amino acid residues, which accumulate a high fraction of the overall mutation load within a protein. Such hotspot mutation residues could have critical functions in regulating cancer-associated cellular processes. After detecting mutation hotspots, the package obtains functional and structural annotations for the affected protein regions as these could aid interpretation of mutation effects. The package is based on the Ensembl version 73 (GRCh37), but it is also flexible with allowing a user to obtain coordinates for different Ensembl releases via the BiomaRt package. The package can be run with the default options using the following command:

```
hotspot_mutations <- DominoEffect(mutation_dataset = TestData, gene_data = DominoData, snp_data = SnpData)
```

Calling package without any parameters will use the same loaded datasets:

```
hotspot_mutations <- DominoEffect()
```

An example mutation set is provided as an object data(TestData, package = “DominoEffect”). It contains mutations reported in a sequencing study for the thyroid cancer (Integrated genomic characterization of papillary thyroid carcinoma, Cell 2014 159(3):676-90, Cancer Genome Atlas Research Network). The mutation\_dataset should provided by the user should have the same columns:

```
data("TestData", package = "DominoEffect")
```

| Ensembl\_gene\_id | Protein\_residue | Original\_aa | Mutated\_aa | Patient\_id | Genomic\_coordinate | Original\_base | Mutated\_base |
| --- | --- | --- | --- | --- | --- | --- | --- |
| ENSG00000204518 | 91 | D | D | TCGA-V4-A9F1-01A-11D-A39W-08 | 1:12711246 | C | T |
| ENSG00000204518 | 112 | R | W | TCGA-V4-A9EK-01A-11D-A39W-08 | 1:12711307 | C | T |
| ENSG00000144452 | 1036 | Q | E | TCGA-WC-AA9E-01A-11D-A39W-08 | 2:215865502 | G | C |
| ENSG00000179869 | 875 | Q | L | TCGA-V4-A9EK-01A-11D-A39W-08 | 7:48311887 | A | T |
| ENSG00000167972 | 689 | D | D | TCGA-YZ-A985-01A-11D-A39W-08 | 16:2347526 | G | A |
| ENSG00000167972 | 155 | R | Q | TCGA-YZ-A985-01A-11D-A39W-08 | 16:2373673 | C | T |

Results of the analysis will be saved as a file Protein\_hotspot\_residues.txt in the working directory at it will contain positions of hotspot residues as well as information on the protein region affected by the hotspot. We recommend to further use one or more of the tools that prioritizes mutations with a likely deleterious effect and to use additional resources to filter out common population polymorphisms.

| Gene | Ensembl\_id | Prot\_position | N\_mut | Protein\_funcional\_region | Repres\_tr | Assoc\_unip\_ids | Perc\_region\_1 | Perc\_region\_2 | Adj\_p\_value\_region\_1 | Adj\_p\_value\_region\_2 | Prot\_length |
| --- | --- | --- | --- | --- | --- | --- | --- | --- | --- | --- | --- |
| GNAQ | ENSG00000156052 | 209 | 37 | nucleotide binding: GTP | ENST00000286548 | P50148 | 94.87% | 94.87% | 0 | 0 | 359 |
| GNA11 | ENSG00000088256 | 209 | 34 | nucleotide binding: GTP | ENST00000078429 | P29992 | 94.44% | 94.44% | 0 | 0 | 359 |
| SF3B1 | ENSG00000115524 | 625 | 14 | repeat: HEAT 3 | ENST00000335508 | O75533 | 77.78% | 77.78% | 0 | 0 | 1304 |
| PRMT8 | ENSG00000111218 | 31 | 5 | motif: SH3-binding 1 | ENST00000382622 | Q9NR22 | 100.00% | 100.00% | 0 | 0 | 394 |
| CHEK2 | ENSG00000183765 | 416 | 5 | domain: Protein kinase; region: T-loop/activation segment | ENST00000382580 | O96017 | 55.56% | 55.56% | 0 | 0 | 586 |

## Set options

The package can also be ran by modifying any or all of the options that are discussed in the section below:

```
hotspot_mutations <- DominoEffect(mutation_dataset, gene_data, snp_data, min_n_muts, MAF_thresh, flanking_region, poisson.thr, percentage.thr, ratio.thr, approach, write_to_file)
```

The analysis has several parameters that define what a hotspot residue is. The default values are suggested below, but these can all be easily modified. As a recommendation, we provide values that we found useful when analyzing the pan-cancer dataset, but for smaller cohorts we strongly suggest these to be relaxed. For instance, for smaller cohorts to set: min\_n\_muts to 3. We recommend the user to try different parameters and if they allow shorter windows around the searched residues to increase the percentage threshold (which defines a minimum fraction of mutations within a window that need to map to a single residue). It is also possible to replace the percentage threshold with a parameter that defines overrepresentation compared to the expected number of mutations at a single residue. Details below.

Define how often a mutation needs to occur on a specific amino acid to be considered as a possible hotspot residue.

```
min_n_muts <- 5
```

Threshold for the minoar allele frequence in the population above which a variant is considered a common variant and will not be assessed as a potential hotspot mutation.

```
MAF.thr <- 0.01
```

Size of the sequence region (in amino acids) to which the frequency of the muation is compared to. We recommend asking for the hotspot to be significant within windows of different lengths, but it is also possible to use a single window.

```
flanking_region <- c(200, 300)
flanking_region <- c(300)
```

Statistical threshold for the residues with frequent mutations that should be considered a protein hotspot. The value defines the adjusted p-values after performing a Poisson test and Benjamini-Hochberg correction for multiple testing.

```
poisson.thr <- 0.01
```

A fraction of mutations within the window that need to fall on a single residue in order for it to be classified as a hotspot.

```
percentage.thr <- 0.15
```

Requirement that a number of mutations on a single residue should exceed what would be expected by chance given a background mutation rate in the window (i.e. the surrounding region).

```
ratio.thr <- 40
```

Hotspots are always filtered to include only residues that are significant according to the defined p-value threshold (i.e. adjusted Poisson p-value: poisson.thr above). Additionally, the parameter ‘approach’ defines whether a percentage or overrepresentation should be assessed to define if the residue accumulates a high fraction of mutations within the window. The options are to set the ‘approach’ parameter to : use “percentage”, “ratio” or “both”. When “percentage” is used the approach checks for the percentage.thr, when “ratio” it cheks for ratio.thr and when it is defined as “both” than it checks both percentage.thr and ratio.thr. If the user considers the p-value threshold to be sufficient, they can set percentage.thr and ratio.thr to zero.

```
approach = "percentage"
```

If the write\_to\_file is set to “YES” the result object ‘results\_w\_annotations’ will be saved in the working directory as a file ‘Protein\_hotspot\_residues.txt’. The default is “NO”.

```
write_to_file = "YES"
```

## Analysis

```
hotspot_mutations <- DominoEffect(mutation_dataset = TestData)
```

It is also possible to run separately the function that identifies hotspot mutations. The function: identify\_hotspots() can be ran on any unique identifiers, but the same column names as in the example DominoData should be preserved. If using different Ensembl releases, the user should provide a table of the same format, and with the same column names (for this we recommend using online Ensembl BioMart or the R package biomaRt). Ensembl identifiers are necessary for obtaining protein sequences in the function: map\_to\_func\_elem().

```
hotspot_mutations <- identify_hotspots(mutation_dataset = TestData, gene_data = DominoData, snp_data = SnpData, min_n_muts = 5, MAF_thresh = 0.01, flanking_region = c(200, 300), poisson.thr = 0.01, percentage.thr = 0.15, ratio.thr = 45, approach = "percentage")
```

If desired, the user can then annotate the identified hotspots using information from the UniProt/Swiss=Prot knowledgebase. Hotspots are identified on the Ensembl sequences. To make sure there are no any discrepancies, during this step sequences are retrieved from the UniProt KB and Ensembl Biomart and then the the Ensembl segment with the hotspot residue (15 aa) is aligned to the UniProt sequence. If different ensembl release than the default is used, two functions should be ran separately and a host address for the respective Ensembl release should be specified in the call to the function: map\_to\_func\_elem(). For instance, for the current release: ens\_release=“www.ensembl.org” instead of ens\_release = “73”.

```
results_w_annotations <- map_to_func_elem(hotspot_mutations, write_to_file = "NO", ens_release = "73")
```

## MutationData, DominoData, and SnpData

As input data the package needs these three datasets. We provide a small example mutation data set TestData (see above).

DominoData (that is used for gene\_data) contains basic information for the genes in the Ensembl version 73. This includes: Ensembl gene identifier, Representative transcript identifier (i.e. a transcript with the longest protein coding sequence), cDNA\_length of the representative transcript, Gene\_name and Associated UniProt identifiers.

The required format of the gene\_data is the following:

| Ensembl\_gene\_id | Representative\_tr | cDNA\_length | Gene\_name | Uniprot\_id |
| --- | --- | --- | --- | --- |
| ENSG00000256574 | ENST00000553795 | 987 | OR13A1 | Q8NGR1 |
| ENSG00000127720 | ENST00000248306 | 1812 | METTL25 | Q8N6Q8 |
| ENSG00000109819 | ENST00000264867 | 2397 | PPARGC1A | Q9UBK2 |
| ENSG00000161057 | ENST00000435765 | 1302 | PSMC2 | P35998 |
| ENSG00000237787 | ENST00000446603 | 303 | C3orf79 | P0CE67 |
| ENSG00000051596 | ENST00000265097 | 1056 | THOC3 | Q96J01 |

Finally, to exclude common polymorphisms we provide SnpData, a set of SNPs with a population frequency higher than 1% that we obtained from the Ensembl BioMart version 73. The user can also set snp\_data = NULL if they do not wish to include this in the analysis. Preferably, they should however use as comprehensive set of population polymorphisms as possible. The dataset has the following format:

| Chr\_name | Position\_on\_chr | Minor\_allele\_freq |
| --- | --- | --- |
| 1 | 150917624 | 0.0160 |
| 1 | 150936280 | 0.0124 |
| 1 | 169823521 | 0.0261 |
| 1 | 169823718 | 0.3516 |
| 1 | 169823790 | 0.0472 |
| 1 | 17256626 | 0.0124 |

## Additional

If the user is starting from genomic mutations, we are happy to share a perl script we wrote for mapping these to Ensembl protein residues. Please contact the package maintainers.

## Session Info

```
sessionInfo()
```

```
## R version 3.3.3 (2017-03-06)
## Platform: x86_64-apple-darwin13.4.0 (64-bit)
## Running under: macOS  10.13.2
## 
## locale:
## [1] C
## 
## attached base packages:
## [1] stats4    parallel  stats     graphics  grDevices utils     datasets 
## [8] methods   base     
## 
## other attached packages:
## [1] Biostrings_2.42.1   XVector_0.14.1      IRanges_2.8.2      
## [4] S4Vectors_0.12.2    BiocGenerics_0.20.0 biomaRt_2.30.0     
## [7] DominoEffect_0.99   knitr_1.15.1       
## 
## loaded via a namespace (and not attached):
##  [1] Rcpp_0.12.8          AnnotationDbi_1.36.2 magrittr_1.5        
##  [4] zlibbioc_1.20.0      bit_1.1-12           highr_0.6           
##  [7] stringr_1.2.0        blob_1.1.0           tools_3.3.3         
## [10] data.table_1.9.6     Biobase_2.34.0       DBI_0.5-1           
## [13] htmltools_0.3.5      yaml_2.1.14          bit64_0.9-7         
## [16] rprojroot_1.2        digest_0.6.10        assertthat_0.1      
## [19] tibble_1.2           bitops_1.0-6         RCurl_1.95-4.8      
## [22] memoise_1.1.0        evaluate_0.10        RSQLite_2.0         
## [25] rmarkdown_1.8        stringi_1.1.2        backports_1.1.0     
## [28] XML_3.98-1.5         chron_2.3-47
```
